# Acute traumatic coagulopathy among major trauma patients in an urban tertiary hospital in sub Saharan Africa

**DOI:** 10.1186/1471-227X-12-16

**Published:** 2012-11-14

**Authors:** Erick Mujuni, Robert Wangoda, Peter Ongom, Moses Galukande

**Affiliations:** 1 Surgery Department, College of Health Sciences, Makerere University, Mulago Hill Road, P. O. Box 7072, Kampala, Uganda; 2 Surgery Department, Mulago National Referral Hospital, Mulago Hill, P. O. Box 7051, Kampala, Uganda

**Keywords:** Acute traumatic coagulopathy, PT/PTT, Major trauma

## Abstract

**Background:**

Mortality from trauma remains a major public health issue as it is the leading cause of death in persons aged 5 to 44 years .Uncontrolled hemorrhage and coagulopathy is responsible for over 50% of all trauma related deaths within the first 48hrs of admission. Coagulation profiles are not routinely done among trauma patients in resource limited settings and there is a paucity of data on acute traumatic coagulopathy (ATC) in sub Saharan Africa. The study was conducted to evaluate the prothrombin time and partial thromboplastin time (PT/PTT) as predictors of mortality and morbidity among major trauma patients.

**Methods:**

A prospective cohort study was carried out, in which major trauma patients admitted in A&E department between December 2011 to April 2012 were recruited. Five (5) mls of venous blood was drawn from a convenient vein within 10 minutes of the patient’s arrival at A&E for analysis of PT/PTT. Patients were stratified into two groups by the presence/absence of coagulopathy then followed up for a 2 week period for morbidity and mortality.

**Results:**

A total of 182 major trauma patients were recruited; 149 (81.9%) were males, the mean age was 29.5 years (SD 9.8). Prevalence of coagulopathy was 54% (98/182). The mean ISS for the ATC group was 36.9 and the non ATC group was 26.9 (p=0.001). Patients with ATC stayed longer in hospital 11.24 days than non ATC patients 8 days (p=0.001). ATC was strongly associated with ARI (p= 0.003). Mortality was more in the ATC group 29 deaths compared to 9 deaths in the non ATC group. PTT was a strong independent predictor of mortality.

**Conclusion:**

A significant proportion of major trauma patients were coagulopathic. Initial coagulation profile is useful in predicting outcomes for major trauma patients.

## Background

Globally trauma is the leading cause of death in persons aged 5 to 44 years [1] and accounts for approximately 10% of all deaths in general [2]. In Uganda more than 25% of all deaths recorded in urban areas are due to trauma [3,4]. Despite substantial improvement in acute trauma care, uncontrolled haemorrhage is responsible for over 50% of all trauma-related deaths within the first 48 hours after admission [5].

To date, six key initiators of coagulopathy in trauma have been described as tissue trauma, shock, hemodilution, hypothermia, acidemia and inflammation [6,7]. Acute traumatic coagulopathy is known to occur in about 28% to 34% of patients with multiple injuries [7]. Other studies have all shown in small cohorts of patients that both civilian and combat trauma is associated with coagulation and fibrinolytic derangements [5,8,9]. Patients who arrive in the emergency department with a coagulopathy are three to four times more likely to die and eight times more likely to die within the first 24 hours [6,7,10,11].

Coagulopathy remains an independent predictor of death in multivariate analyses including injury severity and degree of shock, although there is clearly some interdependence between these variables [10]. PTT is a better predictor of mortality than PT [10]. Coagulopathy on admission is not restricted to mortality only but also associated with other poor outcome of trauma like acute renal injury, acute lung injury, increased transfusion requirements, and long hospital stays [6,7,12,13].

ATC instead of being a dysfunction of the coagulation proteases, it appears to be due to activation of anticoagulant and fibrinolytic pathways [6,7,14]. Most recently, Brohi [6,7] emphasized the role of hypoperfusion for the initiation of ATC. At this center and perhaps similar to many others in sub Saharan Africa, coagulopathy screening are not routinely done due to costs among other reasons. Thus, early recognition accompanied by adequate and aggressive management of ATC they may substantially reduce mortality and improve outcomes in severely injured patients is missed. The purpose of this study therefore was to determine the prevalence of ATC among major trauma patients in a low resource context and to assess the predictive value of the initial coagulation profile (PT/PTT) on related outcomes.

## Methods

### Study design

A prospective cohort study of major trauma patients.

Ethical clearance was obtained from Makerere University College of Health Sciences (MakCHS) Ethics and Research Committee prior to commencing the study.

### Study setting

The study was conducted in the A & E department of Mulago National Referral Hospital situated in Kampala city between December 2011 and April 2012. Kampala is the largest city and capital of Uganda with estimated a population of 1,659, 600 in 2011 [15]. The main mode of transport at Kampala is commuter taxis (15-seater minibus used as public transport) and “Boda-bodas” (local motorcycle transportation) also a popular mode of transport. Deaths from road traffic accidents are on the rise from 778 in 1990 to 2,034 in 2004, while road accidents rose to 19,528 in 2006 from 5,674 in 1990.

The A & E department of Mulago hospital is a fully fledged unit with medical and surgical emergency wings, two operating rooms, an x ray facility, ultra sound facility, resuscitation room with three beds, and a 26-bed holding emergency ward. Adjacent to it are the blood bank, hematology, microbiology and clinical chemistry laboratories. The A and E department is open 24hrs a day and is headed by a consultant surgeon, who leads a team of physicians, internal medicine and surgeons residents/trainees, medical officers and paramedics, nurses, support staff and volunteers. Blood products such as whole blood are normally in short supply owing to very high demand and relatively few donors.

On arrival at the A and E department, trauma patients were triaged and transferred to the examination rooms where they were immediately attended to by doctors who instituted management after history taking and examination. Resuscitation took precedence throughout the above protocol. Patients for operative management were immediately taken to the adjacent casuality operating theatre, while those for observation and further investigations were admitted to emergency surgical ward for up to 24hrs (maximum), before onward transfer to the admitting firm or one of the specialized surgical units.

On average the A and E unit saw about three patients with major trauma daily. Patients transferred from A&E were followed up in the ICU, or appropriate surgical wards.

### Eligibility criteria

We included all patients with major injuries to head and neck, face, thorax, abdomen, extremities, external (surface) with ISS>15. We excluded patients who received more than 2 litres of crystalloids before admission, patients who received blood transfusion before admission, Patients on anticoagulants and those with known co morbidities such as liver diseases/renal failure.

### Study population

All patients with major trauma with ISS>15 admitted at accident and emergency department during the study period and found to meet the eligibility criteria and consented for participation in the study were consecutively recruited into the study.

The outcome variables included; Mortality, renal dysfunction, LOS, PT and PTT.

The sample size was 176 (88 in each group) calculated using the formula for null hypothesis of sample size for two proportions [16,17].

### Procedures

In A&E all major trauma patients were identified during primary survey by severity of injury to the following body areas; head & neck, face, abdomen, thorax, extremities and external surfaces.

For all major trauma patients; 5mls of venous blood were drawn from a convenient vein within 10 minutes of arrival during primary survey of the patient. The sample was placed in a citrated laboratory specimen bottle tube and sent immediately to the laboratory for analysis of PT/PTT which was done within 3 hours (diagnostic thresholds PT >18s, and PPT >36s). Abbreviated injury score (AIS90/ISS) was used to score the severity of injuries of the patients. Other measurements; core body temperature using rectal temperature, blood pressure, pulse rate, respiratory rate and blood for renal function test as baseline were taken during primary survey of the patient.

### Patient identification

The demographic details of the patients were collected using structured/coded interviewer administered questionnaire after primary survey when the patient was already resuscitated and stabilized.

### Patients follow up

Recruited patients were followed up and reviewed on days 0, 2, 6, and 14 while in surgical units. Clinical outcome variables were determined and recorded during these periods. These included blood transfusion requirements (using standard protocols for transfusing blood and blood products in trauma patients). Only whole blood and fresh frozen plasma were available for use. The trigger HB was considered to be less than 99%. Acute renal dysfunction using RFT (BUN/Creatinine >20 as a dysfunction), length of hospital stay (LOS) and death (mortality). Maximum follow-up time was 2weeks. Patients discharged during this period was deemed to be survivors.

### Data analysis

Data was entered into Epi data version 5.3.2, cleaned and coded, then exported to SPSS version 14 for analysis. ATC was defined as PT >18s or PTT >36s. Variables were summarized into means, medians, percentages and proportions. Under the Shapiro-Wilk test/column big valve was greater than 0.005 suggesting participants were normally distributed. The multiple logistic regression analysis was used to ascertain the association between the initial coagulation parameters and overall hospital mortality. Kaplan Meier method was used for survival analysis; log rank test was used to determine the difference between the two survival curves for patients with normal versus abnormal PT/PTT to ascertain if it was statistically significant. The risk ratios (RR) were used as a measure of association of the effect of the predictors on the mortality and morbidity.

## Results

A total of 186 major trauma patients were recruited into the study through the A&E department between December 2011 and April 2012. Their initial coagulation profiles PT/PTT were determined; they were followed up for two weeks to determine their early outcomes. Of these, 4 (2.2%) patients were lost from the study; 3 (1.6%) patients were run away cases and 1 (0.6) patient was transferred to another hospital.

Therefore 182 patients with major trauma were analyzed; 99 (54.4%) patients were coagulopathic and 83(45.6%) patients were non coagulopathic (p=0.017). 149 (81.9 %) were male and 33 (18.1%) were females giving a male to female ratio of 4.5:1.

The age range was 1 to 88 years with a mean of 29.5 years (SD 9.8). There was no significant difference in mean age between the ATC group (29 years) and non-ATC group (30 years) (p=0.375). The majority of patients had primary level education 124 (68.1%), followed by secondary& tertiary education 49 (27.5%), no formal education were 8(4.4%).

On occupation basis “Boda boda” riders (local motorcycle transportation) were the majority among major trauma patients 70 patients (38.5%) followed by peasants & business 89 (48.9%), students were 18 (9.9%) and 5 patients who were employed/salaried (2.7%).

The commonest mode of injury was Road Traffic Crashes (RTC) 118 patients (64.8%), followed by assault 60 patients (32.9%), burn and fall each 2 patients (2.2%). Blunt injury was the commonest 163 (89.6%), then penetrating injury 19 (10.4%) (Table 1).

**Table 1:** Demographics and clinical characteristics of patients with ATC versus non ATC

**Characteristic**	**ATC group**	**Non-ATC group**	**P value**
	**n (%)**	**n (%)**	
**Age** Mean	28.9 (54.4)	30.2 (45.6)	0.375
SD	9.85		
**Sex**			
Male	79 (79.8)	70(84.3)	0.429
Female	20 (20.2)	13 (15.7)	
**Occupation**			
Employed/Salaried	3 (3.0)	2 (2.4)	
Student	10 (10.1)	8 (9.6)	
Peasant& business	43 (43.4)	46 (55.4)	0.593
Boda boda riders	43 (43.4)	27 (32.5)	
**Level of education**			
No education	5 (5.1)	3 (3.6)	
Primary education	63 (63.6)	61 (73.5)	0.308
Secondary &tertiary	31 (31.3)	19 (22.9)	
**Cause of injury**			
RTC	68 (68.7)	50 (60.2)	
Assault	30 (30.3)	30 (36.1)	0.356
Fall	0 (0)	2 (2.4)	
Burn	1 (1.0)	1 (1.2)	
**Type of injury**			
Blunt force	91 (91.9)	72 (86.7)	
Penetrating	8 (8.1)	11 (13.3)	0.517
**ISS mean score (SD)**	36.9(14.1)	26.9(10.9)	0.001
**Time delay**			
**Mean time(SD) hrs**	4.5(4.2)	3.6(1.9)	0.05
1-6h time delay	93 (94)	75 (91)	0.152
7-12h time delay	6(6)	4(5)	
13-24h time delay	0(0)	3(4)	
**Length of stay (LOS)**			
**LOS mean (SD) days**	11.2(3.5)	8.2(4.2)	0.001
**Blood transfusion (whole blood)**			
Transfused	41	27	0.018
**Acute renal injury**			
ARI	25	7	0.003

The average interval between the time of injury and admission to the A & E department of Mulago hospital for patients with major trauma was 4 hours with a range of 0.5 hours to 24hrs (SD 3.2 CI 3.5-4.5). For patients injured within Kampala the mean time was 2 hours, and those outside Kampala was 5 hours. The commonest mode of transportation was police patrol pick up trucks 155/182 (91%). Patients with ATC spent a longer time between injury and arrival at A & E than non-ATC patients (p=0.05). The mean ISS was 32 (SD 14 CI 30–34) among major trauma patients. Patients with ATC had a higher mean ISS than patients with non-ATC (p=0.001).

ATC patients stayed longer in the ward 11 days than non-ATC patients 8 days (p=0.001). ATC was strongly associated with ARI (p=0.003) and was also associated with increased transfusion requirements though was not statistically significant (p=0.179).

A total of 67 (37%) patients with major trauma had elevated PTT. Among major trauma patients a total of 99 (54%) had coagulopathy and 83 (46%) had no coagulopathy. Prevalence of coagulopathy in the study population was 54%.

The overall mortality in study population was 38 (20.9%).Mortality was more in the ATC group 29 (29.3%) p= 0.002. The incident risk ratio of dying was more in the ATC group (IRR 2.7) than in the non-ATC group (p=0.001) (Table 2).

**Table 2:** Overall coagulopathy and mortality among trauma patients

**Category**	**ATC**	**Non ATC**	**p value**
	**N (%)**	**N (%)**	
Patients (n)	99	83	-
Mortality	29	9	0.002
Survived	70	74	-
IRR	2.70(1.28-5.71)	-	0.001
PT Elevated	82 (45)	-	-
PT Normal	100 (55)	-	-
A PTT Elevated	67 (37)	-	-
A PTT Normal	115 (63)	-	-
**Time delay**	**Died**	**Survived**	
	**n (%)**	**n (%)**	
1–6 h	38 (100)	130(91)	0.156
7-12h	0	10(7)	
13-24h	0	3(2**)**	
**OR (CI)**			
1-6h	1.2 (0.9-1.7)		0.236
7-12h	1.5 (0.4-5.3)		
13-24h	0		

**Figure 1:**
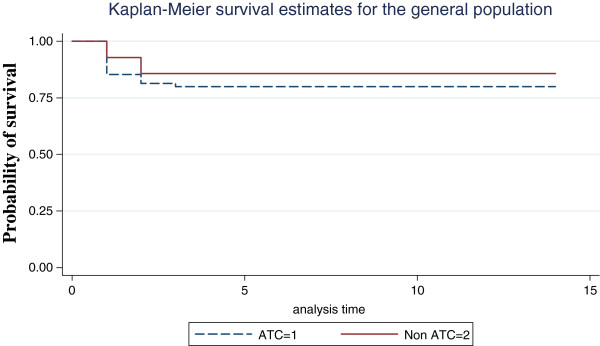
Probability of survival in the study population for PT.

Kaplan Meier curve and log rank test demonstrated significant difference in survival between ATC and non-ATC group (p=0.001) (Figure 1).

**Figure 2:**
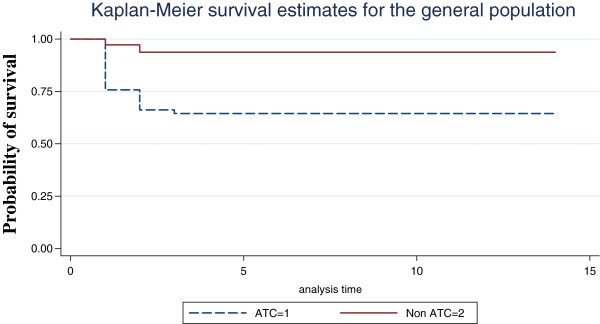
Probability of survival in the study population for aPTT.

Logistic regression analysis has shown PTT to be strong independent predictor of mortality even in the presence of other predictors of mortality (Figure 2).

## Discussion

We set out to determine the prevalence of acute traumatic coagulopathy among major trauma patients and was found to be 54%. This prevalence is much higher than what has been reported in other studies outside Uganda ranging from 24 to 34% [6,7,10,11]. This could be due to the fact that the design of this study included only patients with major trauma while some of other studies included all trauma patients (minor and major). The average time of injury to admission was 4 hours, compared to less than 70minutes in other contexts with well-functioning ambulance system and infrastructure [7,10] perhaps this time delay and other factors like hypothermia could have contributed to the high prevalence.

In addition, numerous authors have documented that cohorts of head injury patients have a high prevalence of coagulation abnormalities [18-22]. The fact that some of the patients had head injuries certainly contributes to this picture of coagulopathy.

The mode of transport from the injury scene to hospital was inadequate or inappropriate, as most patients 155(90.7%) were brought by police patrol pick-up trucks and other cars which are not fitted with ambulance facilities hence didn’t get any pre hospital resuscitation, this is a common occurrence in most resource poor settings.

### Pre hospitalization delay and length of hospital stay

The mean time from injury to arrival at hospital was 4 hours (with a range between 0.5 hours to 24 hours). For patient within Kampala (10-15km radius) it took 2 hours and those outside Kampala was 5 hours similar to findings from the other studies done in Kampala [23-25].

The average time from injury to admission for coagulopathic patient was 4 hours and 3.6 hours for non coagulopathic patients (p=0.05), time of injury to admission could have contributed to the outcome in major trauma patients.

Duration of injury before admission is still high (therapeutic vacuum) as compared to other trauma centers [7,10].

For coagulopathic group the mean LOS was more in the non coagulopathic group (p=0.001). Several investigators have reported significance increase in the LOS in trauma patients with coagulopathy [6,7,10,11].

However, the analysis for LOS in our study was done only for trauma patients who survived i.e. 144 (79%) patients. A considerable number of major trauma patients died within the first day 28 (15.4%) and second day 6 (3.3%) from admission with an overall mortality of 20.9%. Patients with coagulopathy who survived had longer LOS in either ICU or on the general wards as they needed more clinical management support.

### Acute renal injury (ARI) and blood transfusion requirements

There were more ARI in the coagulopathic group 25 (25.3%) patients than in the non coagulopathic group 7 (8.4%) (p=0.003). This is comparable with other studies done outside Uganda on ATC [6,7].

However the exact relationship between ARI and ATC needs to be further investigated.

There was no strong association between blood transfusion requirements and coagulopathy. A total of 41(41.4%) of patients with coagulopathy were transfused and 27 (32.5%) of patients without coagulopathy were transfused with different blood products (p=0.179). Increased transfusion requirements in major trauma patients were probably due to two events; blood loss at the scene (event) and continue loss secondary to coagulopathy.

Lack of significant difference in our study could be because of non compliance to standard protocol as far as blood transfusions practices is concerned in our setting because in part there is frequently inadequate supply of blood during the day but more so at night.

### Mortality

The overall mortality was 38(20.9%), this is higher mortality than what has been reported in developed world. Kirya reported a mortality of 39(26%) among major trauma patients in a study of outcome of major trauma patients at Mulago hospital 10 years ago [24].

Other studies reported an overall mortality among major trauma patients ranging from 15% to 20%, however these studies where done in high resourced trauma centres [6,10,11].

The mortality was more in the coagulopathic group 29(29.3%) than in the non coagulopathic group 9(12.2%) P=0.002, this is comparable with other studies [6,10,11].

In this study, coagulopathy was a strong predictor of mortality in major trauma patients (IRR 2.7 95% CI 1.3 - 5.7, p = 0.001) and a predictor of morbidity (longer length of stay).

The Kaplan-Meier survival curves suggest a significant difference in probability of survival between patients with elevated PTT and those with normal (p=0.001). Most deaths resulting from elevated PTT occur early in the hospital stay, with the probability of survival paralleling between the two groups as time goes on. Thus PTT was a strong predictor of outcome than PT. Multiple regressions showed PTT, systolic BP, GCS were the variables that influenced outcome the most.

The ability to determine whether the trauma patient at admission is coagulopathic or not is a single most important predictor of outcome. This is comparable with other studies on ATC [6,7,10]. This study was not without limitations; perhaps additional variables such as INR (International Normalized Ratio), temperature (to detect hypothermia), metabolic acidosis and fibrin break down products would have added valuable information to ascertain coagulopathy. So is the lack of blood products that is encountered often times in the late night hours we did not catergorise which patient came at night or during the day. With longer pre hospital times we may be seeing a mixture of trauma induced coagulopathy (TIC) and ATC rather than ATC alone. Regardless of whether ATC or TIC a large number of patients presented with deranged coagulation.

## Conclusion

In the quest to improve major trauma outcomes in resource limited environments, we suggest that coagulopathy assessment is done routinely in trauma care practice. In addition, affordable and effective ways to assess reverse or prevent coagulopathy in early trauma stages should be investigated further.

## Competing interest

The authors declare that they have no competing interests.

## Authors’ contribution

EM originated the concept and wrote the first draft. MG, RW, and PO contributed to writing the manuscripts and performed critical reviews for intellectual content. All authors read and approved the final manuscript.

## Pre-publication history

The pre-publication history for this paper can be accessed here:

http://www.biomedcentral.com/1471-227X/12/16/prepub
